# Doctors in Medical Data Sciences: A New Curriculum

**DOI:** 10.3390/ijerph20010675

**Published:** 2022-12-30

**Authors:** Sylvain Cussat-Blanc, Céline Castets-Renard, Paul Monsarrat

**Affiliations:** 1 Artificial and Natural Intelligence Toulouse Institute ANITI, 31013 Toulouse, France; 2 Institute of Research in Informatics (IRIT) of Toulouse, CNRS—UMR5505, 31400 Toulouse, France; 3 Civil Law Faculty, University of Ottawa, Ottawa, ON K1N 6N5, Canada; 4 RESTORE Research Center, Université de Toulouse, INSERM, CNRS, EFS, ENVT, Batiment INCERE, 4bis Avenue Hubert Curien, 31100 Toulouse, France; 5 Department of Oral Medicine, Toulouse University Hospital (CHU de Toulouse), CEDEX 9, 31062 Toulouse, France

**Keywords:** machine learning, data science, medical education

## Abstract

Machine Learning (ML), a branch of Artificial Intelligence, which is competing with human experts in many specialized biomedical fields and will play an increasing role in precision medicine. As with any other technological advances in medicine, the keys to understanding must be integrated into practitioner training. To respond to this challenge, this viewpoint discusses some necessary changes in the health studies curriculum that could help practitioners to interpret decisions the made by a machine and question them in relation to the patient’s medical context. The complexity of technology and the inherent criticality of its use in medicine also necessitate a new medical profession. To achieve this objective, this viewpoint will propose new medical practitioners with skills in both medicine and data science: the Doctor in Medical Data Sciences.

## 1. Context

The tremendous increase in the amount and diversity of data in medicine requires the development of new tools capable of integrating these data for the purpose of personalized medicine [1,2]. Machine Learning (ML), a branch of Artificial Intelligence (AI), is competing with human experts in many specialized biomedical fields (e.g., analysis of ECG, in imaging, dermatology, histopathology) and provides an opportunity for an in-depth understanding of multidimensional sources of biopsychosocial data [1,2]. With the development of AI techniques to analyze complex heterogeneous data, virtual assistants will enter the daily routine of future medical doctors. The current applications of AI range from the generation of reports to theranostic assistance in a wide range of medical fields [3,4]. Many future applications might also be of interest to emerging countries in which access to specialists and diagnostic devices is limited [5]. In contrast with this data-driven medicine, maintaining the human focus of healthcare is crucial. The practitioner must remain the guardian of medical decisions regarding technology [4,6]. There is a urgent need to develop professional competencies for the use of AI-based tools, promoting AI in shared decision-making models, develop human-centric AI algorithms and create appropriate educational frameworks [7]. Therefore, two cases can be identified:

1. “Off-the-shelf” tools are available. No significant technical knowledge is required but an understanding of the underlying technology is necessary (how to implement these tools in a given clinical case; how to explain and interpret the results). Therefore, it seems essential that each practitioner is able to interpret the decisions made by a machine and question them in relation to the patient’s medical context [4,6,8]. As with any other technological advances in medicine, the keys to understanding must be integrated into practitioner training.
2. To develop customized data analysis pipelines when commercial solutions to a specific medical question do not exist. To achieve this objective, medical practitioners, with double competencies in medicine and data science, should be required in hospitals, within a real data science department.

While many formations currently exist to provide an education to science, technology, engineering, and mathematics (i.e., STEM) students regarding the use of ML in general and, in some cases, within a biomedical context, the health sector lacks specialists that could develop and understand the pros and cons of potential AI-based approaches, and participate in patients’ collegial medical care by rationalizing the use of these technologies.

Consequently, to respond to the challenges induced by the democratization of AI/ML, we suggest some changes in the health studies curriculum and the creation of a new medical specialty, “Doctor in Medical Data Sciences” (MDS). The goal of this AI/ML education is to create a synergy between data scientists and medical doctors to produce, test and deploy better AI-assisted solutions. We will discuss how the current and new curriculum would be impacted by this viewpoint.

## 2. Minimum Common Core AI/ML Understanding for Initial and Continuous Education in Medicine

Equipping the practitioner to understand the complex decisions made by the algorithm [9], which are fraught with consequences, requires a minimum level of training in algorithms (their pros and cons, benefits, and limitations). An in-depth understanding is unnecessary, while cultivating a critical capacity in the subject is essential [6,10].

To this end, the minimal curriculum should include a brief history of AI and ML methods, and a description of the different types of algorithms (e.g., expert systems, decision trees, support-vector machines, forest-based algorithms, and neural networks), as well as their different properties (supervised/unsupervised, online/offline) [1].

**Figure 1:**
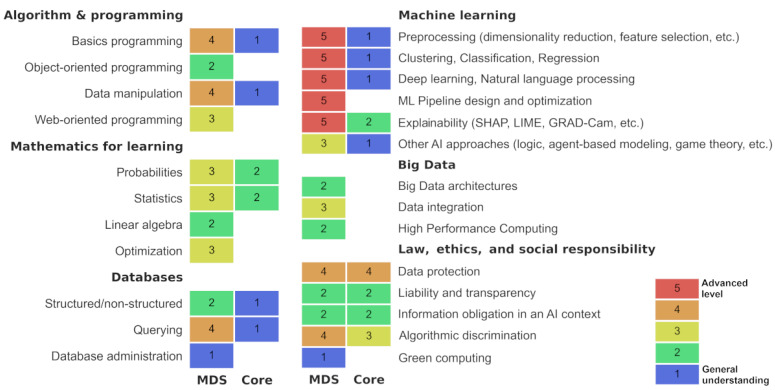
The proposition of a syllabus for Medical Data Scientists (MDS) and common core AI/ML in medical education (Core). While the Core program would concern all MD trainees and continuous education, the MDS specialty must go beyond this, and be built as an applied research program. The syllabus was divided into six categories, with details for each and expected levels. A specific level implies all levels below it.

Future practitioners should understand the entire data analysis pipeline, how and where the data have been stored and annotated, how they are pre-processed and transformed into a dataset, how machine learning algorithms are trained and what biases can be introduced, and how predictions should be interpreted and explained [8,9]. Details are provided in Figure 1.

We recommend active instruction through flipped classrooms to maintain interactivity and discuss situational scenarios. Practical work manipulating ML algorithms with simulators (e.g., simbrain [11]) will provide a better understanding of their inner functioning.

Moreover, ML should be placed in a social context, including the risk of biases (e.g., gender or ethnicity biases of dataset) and reliability (e.g., pixel attack, noise robustness) [5,8,12]. ML should be taught to practitioners as an additional tool to complement or even enhance the “patient-centered” vision of care. Finally, the practitioner must be educated in ethical and legal issues [6,10], especially regarding the massive use of sensitive personal data and algorithmic discrimination. He/she must also respect his/her obligation to inform the patient and obtain free and informed consent, including, in the case of use of ML, predictions, recommendations or assistance in decision-making.

## 3. Proposal of a New Medical Specialty/Profile: Doctor in Medical Data Sciences (MD MDS)

### 3.1. Objectives of the MDS Specialty

This new medical specialty would combine expertise in medicine, computer science and ML. Medical knowledge is mandatory to understand and assess the current state of a patient’s health and requires constant interaction with other clinical specialties. The role of the MDS should be to coordinate and implement data analysis strategies, integrating multiple sources of data, from the electronic medical records, published scientific data or other publicly available data for contextualization (e.g., meteorological data, soil and water pollution and social media). Medical issues could range from a review of radiological acquisitions by deep learning algorithms to an ML-based determination of the best treatment solution for a cancer based on a set of clinical, socio-demographic, and biological data.

### 3.2. MDS, the Head of the Medical Data Sciences Department

The MDS would bridge the gap between data scientists and the medical team, including in multidisciplinary consultation meetings, where he/she would play the key role of data integrator. The MDS should not be considered in isolation but coordinated with the engineers, statisticians, mathematicians, and computer scientists within a new Medical Data Sciences Department, which is fully integrated in the healthcare structures and established in close collaboration with the IT department and the computational infrastructures. The main role of the MDS would then be to coordinate all the actors and projects in this department.

In a nutshell, the MDS should be the cornerstone of data integration in the health infrastructures, as well as a central player in personalized medicine through its abilities (1) to both evaluate and implement new AI-based commercial technology solutions in the field and ensure vigilance, (2) develop new applications based on the needs of medical specialties and patients, (3) implement data analysis strategies to precisely address a patient’s medical situation.

The MDS Department should manage data integration from the locoregional to the international level to develop ML models as accurately as possible. With the advances in transfer and federative learning, the MDS department will play a crucial role in the collective management of AI in medicine.

### 3.3. Training and Required Skills for MDSs

While medical studies can remain unchanged (regardless of the educational system), the MDS requires strong skills in data science (Figure 1). The MDS would be required to extract data from the information system, adequately pre-process them to make them usable to train models, and understand the key differences between state-of-the-art machine learning models such as linear, polynomial and kernel regression (e.g., Support Vector Machines), tree-based approaches (e.g., random forest, XGBoost) or neural networks (e.g., multilayer perceptron, deep learning). The MDS should also master other key notions such as hyperparameter optimization, overfitting, management of dataset imbalance. As most existing techniques are “black-box” approaches, critical analysis competencies would be required to scientifically evaluate the usability of the algorithms and, when possible, help to explain the decisions/results.

Different training programs are already provided in various universities, and it is necessary to build on them [13,14,15]. Ideally, the courses should be applied to the biomedical field. As a guideline, we propose the acquisition of a master’s degree during the first years of medical training and the preparation of a PhD during the internship within the Medical Data Sciences Department, where she/he will participate both in the work of the department and in the progress of the thesis project, directed by an MDS of the department or, ideally, co-directed with a researcher in mathematics or informatics. At the end of her/his training and the obtaining of the PhD, he or she will acquire the title of Doctor of Medicine, specialist in data sciences.

Beyond the functioning of the department itself, there are many opportunities for future employment (clinics, entrepreneurship, pharmaceutical companies, data sciences consultants for medical applications, etc.).

## 4. Conclusions

Regardless of the solution that is chosen, it is undeniable that AI/ML will play a major role in precision medicine. The point here is not to replace the practitioner but to provide him/her with assistance and reinforcement [1,2]. The complexity of technology and the inherent criticality of its use in medicine necessitate a new medical profession: The Doctor in Medical Data Sciences.

The MDS should not be considered alone but with the coordination of mathematicians and computer scientists within a new department, the Medical Data Sciences Department, which is fully integrated with the healthcare structures and interacts with the IT department. This department could function as any hospital department, with beds (i.e., virtual beds) in line with the available human resources (MDS, computer scientists, mathematicians) and computational power. The MDS would also bridge the gap between the industry and the medical centres to foster discussions during project development and produce more adequate solutions.

Finally, the MDS department should manage the local, regional, national and even international integration of data to develop ML models that are as accurate as possible. With advances in transfer and federative learning, the MDS department will play a crucial role in the collective management of AI in medicine.

## Data Availability

Not applicable.

## Abbreviations

The following abbreviations are used in this manuscript:

AI	Artificial Intelligence
ECG	Electrocardiogram
ML	Machine Learning
MDS	Doctor in Medical Data Sciences
STEM	Science, technology, engineering, and mathematics
